# Asymmetric Variate Generation via a Parameterless Dual Neural Learning Algorithm

**DOI:** 10.1155/2008/426080

**Published:** 2008-04-24

**Authors:** Simone Fiori

**Affiliations:** Dipartimento di Elettronica, Intelligenza Artificiale e Telecomunicazioni (DEIT), Università Politecnica delle Marche Via Brecce Bianche, Ancona I-60131, Italy

## Abstract

In a previous work (S. Fiori, 2006), we proposed a random number generator based on a tunable non-linear neural system, whose learning rule is designed on the basis of a cardinal equation from statistics and whose implementation is based on look-up tables (LUTs). The aim of the present manuscript is to improve the above-mentioned random number generation method by changing the learning principle, while retaining the efficient LUT-based implementation. The new method proposed here proves easier to implement and relaxes some previous limitations.

## 1. INTRODUCTION

Random numbers are currently used for a variety of purposes such as: cryptographic keys generation, games, some classes of scientific experiments as well as “Monte Carlo” methods in physics and computer science [1–6]. Standard programming environments are endowed with basic pseudorandom signal generators such as the uniform and the Gaussian ones, while usually the needed distributions are more involved than uniform/Gaussian. A simple example of application is password generation: a random password generator is a software that inputs from a random or pseudorandom number generator and automatically generates a password. An example of application where involved probability distributions are needed is in independent component analysis (ICA, [7]) testing: as the behavior of an ICA algorithm might depend on the statistical distribution of the sources, ICA-algorithm testing tools might require random sequences generators capable of producing random numbers distributed according to involved probability laws.

The principal methods known in the literature to obtain a batch of samples endowed with an arbitrary distribution from a samples batch having a simple distribution are the “transformation method” and the “rejection method” [8]. In the present paper, we focus on the transformation method, which may be well implemented through a tunable neural system, because the availability of a random number source and of a tunable nonlinear system, along with a proper learning procedure, allows obtaining a wide class of pseudorandom signal generators.

**Figure 1:**
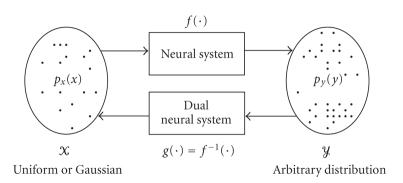
Neural system, neural dual system, input/output sample spaces and their statistical distributions.

A well-known effect of nonlinear neural systems is to warp the statistical distribution of its input. In particular, we assume that the system under consideration has a nonlinear adaptive structure described by the transference *y* = *f*(*x*), where *x* ∈ *𝒳* ⊆ ℝ denotes the system input random signal, having probability density function *p*_*x*_(*x*), and *y* ∈ *𝓎* ⊆ ℝ denotes the output signal, having probability density function *p*_*y*_(*y*), as shown in Figure 1. In the hypothesis that the neural system transference is strictly monotonic, namely *f*′ (*x*) > 0, for all *x* ∈ *𝒳*, the relationship between the input distribution, the output distribution, and the system transfer function is known to be [9]

(1) $$p_{y}(y)={\frac{p_{x}(x)}{f^{\prime }(x)}\;|}_{x=f^{-1}(y)},\;x\in X,$$

where *f* ^−1^(⋅) denotes the inverse of function *f*(⋅). Usually, (1) is interpreted as an analysis formula, which allows computing the output distribution when the input distribution and the system transference function are known. However, the cardinal equation (1) may also be interpreted as a formula that allows for designing the nonlinear system when the distribution *p*_*x*_(⋅) is known and it is desired that the system responds according to a desired distribution *p*_*y*_(⋅). In fact, (1) may be rewritten as the differential equation:

(2) $$f^{\prime }(x)=\frac{p_{x}(x)}{p_{y}(f(x))},\;x\in X.$$

In general, such design operation is rather difficult, because (2) in the unknown *f*(⋅) involves the solution of a nonlinear differential equation, provided that a consistent boundary condition is specified.

In the recent contribution [10], we presented a pseudorandom samples generator based on a nonlinear monotonic neural system, whose transference function is denoted by *f*(⋅), tuned on the basis of the differential equation (2). The cardinal design equation (2) was proposed to be solved via a (relaxation-type) fixed-point algorithm. The key advantages of the method proposed in [10] are as follows. (a) In order to obtain a fully-tunable neural transference function, a look-up-table representation was chosen. It guarantees high flexibility in the shape of the neural transference as well as easiness of representation and handling of the involved quantities. (b) The fixed-point learning algorithm exhibits fast convergence over other possible methods such as the gradient-based one: unlike these methods, the fixed-point learning algorithm does not require the computation of derivatives of the involved functions.

The resulting random-number generation method should be thus read as a two-stage procedure. The first stage consists in solving the cardinal differential equation (2) in the unknown function *f*(⋅), given the distributions *p*_*x*_(⋅) and *p*_*y*_(⋅) as data. The second stage consists in generating input random samples drawn from the distribution *p*_*x*_(⋅), then letting such random samples pass through the learnt nonlinear neural system by computing output values *y* = *f*(*x*). The random samples *y* are assured to be distributed according to the probability density function *p*_*y*_(⋅).

However, we recognized that the method presented in [10] also suffers from some drawbacks, namely the following. (a) For numerical convergence purpose, each step of the fixed-point-type tuning algorithm needed to be followed by some normalization steps. Namely, from (2), it is easily seen that when the function *p*_*y*_(*f*(*x*)) approaches 0, the computation of *f*′ (*x*) becomes ill-conditioned, therefore the quantity *p*_*y*_(*f*(*x*)) was replaced by *p*_*y*_(*f*(*x*)) + *γ* , with *γ* > 0 being a small constant to be properly sized. Also, in order to refine learning, after each iteration step, the solution *f*(*x*) needed to be normalized either by affine scaling, in order to control the range of variable *y*, or by linear scaling in order to match the true value of output distribution moment of preselected order. This, in turn, requires computing in advance the (closed form) moments of interest of the output distribution. (b) In spite of affine scaling, it was not easy to control the range of the output value *y*, as affine scaling does not guarantee convergence in every case of interest, therefore it could not be employed in every case. (c) The developed procedure was customized to generate output distributions that are either symmetric (namely, *p*_*y*_(−*y*) = *p*_*y*_(*y*)) or completely skewed to the right (namely, *p*_*y*_(*y*) = 0, for all *y* < 0) only. Asymmetric or general-shape distributions were not considered.

In the present paper, we consider the problem of extending the previous method to the generation of asymmetric distributions by removing the constraint of symmetry or skewedness to the right. Also, we propose a way to avoid normalization of probability density function. The solution of choice implies a change in the viewpoint of cardinal equation (1): instead of converting formula (1) into the differential equation (2), we convert it into a new differential equation, hereafter referred to as *dual cardinal equation*, which will prove easier to solve and more flexible to use in practice, while retaining the previous numerical representation/advantages. Thus, we will retain the effective numerical representation of the involved quantity already introduced in the works [10, 11], based on the “look-up table” (LUT) implementation of neural activation functions as well as the efficient numerical algorithm to solve the dual cardinal equation. LUTs were proven to provide an efficient way of representing and handling the variables appearing within the devised random number generation algorithm. A prominent advantage of the procedure is the lack of hard computational requirements except for LUT handling, which consists of sorting/searching on lists of numbers and of few simple algebraic operations on numbers.

The effectiveness of the proposed approach will be evaluated through numerical experiments. In particular, the designed experiments followed a logical succession, beginning with a basic assessment of the proposed method when applied to bi-Gaussian distribution, which is then followed by comparably more difficult distributions, namely a generalized Gaussian distribution and an asymmetric Gamma distribution.

The existing method presented in [3] is worth discussing. It concerns a neural-networks-type algorithm to generate random vectors with arbitrary marginal distributions and correlation matrix, based on NORTA method. The “normal-to-anything” (NORTA) method (see, e.g., [12]) is one of the most efficient methods for random vector generation. In [3], a technique was presented to generate the correlation matrix of normal random vectors based on an artificial neural networks approach. The NORTA algorithm works in the following way to generate random samples with prescribed probability density function. First, generate zero-mean unit-variance random samples *x*_*i*_, *i* ∈ {1,…, *Q*}. Then, generate the desired random samples as *y*_*i*_ = *P* ^−1^_*y*_ (Φ(*x*_*i*_)), where Φ(⋅) denotes the cumulative distribution function of a standard normal random variable and *P*_*y*_(⋅) denotes the desired cumulative distribution function, with *P* ^−1^_*y*_ (*u*) = inf {*z* ∣ *P*_*y*_(*z*) ≥ *u*}, *u* ∈ [0 1]. It appears, thus, as a transformation method.

Most of the methods of random vector generation known from the literature impose constraints on the size of the random vectors and many of them are applicable only for bivariate distributions whose components are equidistributed. Conversely, within the NORTA framework, marginal probability distributions for vector components as well as their correlation matrix may be specified. Obtaining the prescribed generated random vector correlation matrix requires solving an involved nonlinear system of equations, which is the most serious problem in this kind of approach. Paper [3] makes use of a multilayer perceptron neural network to estimate correlation matrices of normal random vectors, allowing thus to overcome the analytically involved equations of NORTA algorithm. While the method proposed here is more general than NORTA in the sense that it works for any kind of available generator (not only Gaussian), it is less general in the sense that it does not allow to generate multivariate random variables with prescribed joint statistics. 

## 2. DUAL CARDINAL EQUATION AND ITS NUMERICAL SOLUTION

The present section formalizes the learning problem at hand and illustrates a fixed-point-based numerical algorithm to solve the dual cardinal equation.

### 2.1. Dual cardinal equation and neural system

The key point of the new method consists in learning the inverse function *f* ^−1^(⋅) instead of the function *f*(⋅). As it will be clarified in the next sections, this choice simplifies the learning problem while adding slight computational burden to the usage of the learnt neural system as a generative model.

We denote by $x=g(y)\overset{\text{def}}{=}f^{-1}(y)$ the inverse function of the actual neural transfer function and refer to the new neural system, having *g*(⋅) as transfer function, as the “dual neural system” (shown in Figure 1). The purpose here is to learn a dual neural system that warps *p*_*y*_(⋅) into *p*_*x*_(⋅) under the constraint *g*′ (*y*) > 0, for all *y* ∈ *𝓎*. We denote the interval of interest for the generated random variable as $Y=[\underset{̲}{y}\;\overset{¯}{y}]$. With this hypothesis on the nonlinear dual neural transfer function, the cardinal equation (1) may be rewritten as

(3) $$g^{\prime }(y)=\frac{p_{y}(y)}{p_{x}(g(y))},\;g(\underset{̲}{y})=0,\;\;y\in Y,$$

which will be hereafter referred to as “dual cardinal equation.” It is worth noting that the boundary condition $g(\underline{y})=0$ is completely arbitrary. While there are no theoretical reasons to set the boundary condition in any specific way, the above choice is motivated by the observation that it simplifies the fixed-point adapting algorithm with respect to the previous version proposed in [10].

In general, a closed-form solution to (3) may not be realized, thus we should resort to an iterative learning algorithm to search for a solution. Formally, this means designing an algorithm that generates a succession of functions *g*_*n*_(*y*), *n* ∈ ℕ, whose limit coincides to the solution of (3). A way to generate such a succession is to employ the algorithm:

(4) $$g_{n+1}(y)=\int_{\underline{y}}^{y}\frac{p_{y}(t)dt}{p_{x}(g_{n}(t))},\;n\geq 0,\,\;\;y\in Y.$$

As a figure-of-convergence of learning process, we consider the weighted difference of function *g*(⋅) between two successive iterations, namely,

(5) $$\Delta g_{n}\overset{\text{def}}{=}\int_{Y}\;|\;g_{n}(y)-g_{n-1}(y)\;|\;p_{y}(y)dy,\;n\geq 1.$$

As initial guess, we assume *g*_0_(*y*) = 0, for all *y* ∈ *𝓎*.

After learning an inverse function *g*(⋅), the numerical procedure should calculate the actual nonlinear function *f*(⋅) by numerical inversion. As it will be clarified in the next section, within the framework proposed here, such operation involves a very little computational effort.

### 2.2. Numerical implementation of the learning procedure

From an implementation viewpoint, the algorithm (4) needs to be discretized in order to obtain a version suitable to be implemented on a computer.

We choose to represent function *g*_*n*_(*y*) by a numerical vector: in practice, we suppose the interval Y=[y̲yy¯] of interest to be partitioned into *N* ≥ 1 discrete bins. This gives rise to the vector-type representation **y** ∈ ℝ^*N*+1^ of the support of the output sequence probability density function, where **y** contains *N* + 1 values regularly spaced in *𝓎* with spacing-width denoted as Δ_*y*_. Then *g*_*n*_(*y*) may be represented by a numerical vector **g**_*n*_ ∈ ℝ^*N*+1^ and the neural input-output transference is now represented by the discrete relationship (**g**, **y**) ∈ ℝ^*N*+1^ × ℝ^*N*+1^, namely, a numerical *look-up table*. The entries of a vector **g**_*n*_ may be denoted by an extra footer, that is, by *g*_*n,k*_, with *k* ∈ {0, 1,…, *N*}. The interval Δ_*y*_ relates to the integer *N* and may be defined as $\Delta_{y}\overset{\text{def}}{=}(\overset{¯}{y}-\underset{̲}{y})/N$.

In order to translate the learning rule (4) into a version suitable to numerical representation, we should consider the inherent limitations of numerical integration of differential equations. The following notes are worth taking into account. (a) *Output support selection*: the ultimate purpose of the random number generation method under construction is to generate random samples with desired probability distribution *within a range of interest*, namely, with values within an interval that is deemed suitable for the purposes that random samples generation is launched for. Therefore, the output range Y=[y̲yy¯] is to be freely selected according to the needs the random samples are to be generated for. Then, the above-mentioned vector **y** has entries *y*_*k*_ computed as $y_{k}=\underline{y}+k\cdot \Delta_{y}$, with *k* ∈ {0, 1,…, *N*}. (b) *Input support selection*: in order to prevent the denominator of the quantity *g*′_*n*+1_(*y*) in (4) to become too close to zero, a sensible choice is to carefully select the support *𝒳*. As in this paper we consider the input probability density function to be either (symmetric) Gaussian or uniform, we set *𝒳* = [−*R*_*x*_ *R*_*x*_], with *R*_*x*_ > 0. The value of constant *R*_*x*_ is to be selected in such a way that *p*_*x*_(*R*_*x*_) ≫ 0. It is worth recalling that the support of the input distribution may be arbitrarily selected as it does not affect the support of the output distribution. (c) *Iterative range scaling*: after each learning step, an affine normalization operation is performed, that linearly scales the entries of the putative solution **g**_*n*_ so that *g*_*n*,0_ = −*R*_*x*_ and *g*_*n,N*_ = *R*_*x*_.

**Figure 2:**
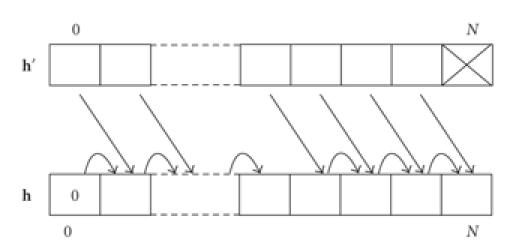
Behavior of the “cumsum” operator for look-up tables.

In order to describe the numerical learning algorithm, the following operators are defined for a generic look-up table (**h, y**) ∈ ℝ^*N*+1^ × ℝ^*N*+1^:

(6) $$\begin{array}{c} \text{cumsum}(h{)}_{0}=0\;,\;\;\;\text{cumsum}(h{)}_{k}\overset{\text{def}}{=}\overset{k-1}{\underset{i=0}{\sum }}g_{i}\Delta_{y}\;,\\ \text{affscale}\{h;a,b{\}}_{k}\overset{\text{def}}{=}a+\frac{\left(h_{k}-\text{min}\{h\})(b-a\right)}{\text{max}\{h\}-\text{min}\{h\}}, \end{array}$$

where the subscript *k* denotes the *k*th entry of the vectors (**h**) and {**h**; *a, b*}. The behavior of the “cumsum” operator is illustrated in Figure 2, which also provides a visual representation of look-up tables. In practice, the considered numerical version of the learning rule (4) writes

(7) $$\begin{array}{ll} (A0)\;\; & g_{0}\,:=0,\\ (A1)\;\; & {g\prime }_{n+1}:=\frac{p_{y}}{p_{x}(g_{n})},\;n\geq 0,\\ \;(A2)\;\; & g_{n+1}:=\text{cumsum}\{{g\prime }_{n+1}\}\;,\\ (A3)\;\; & g_{n+1}:=\text{affscale}\{g_{n+1};-R_{x},R_{x}\}\;, \end{array}$$

where symbol := denotes vector values assignment and **p**_*y*_ denotes the vector of *N* + 1 entries containing the values of *p*_*y*_(⋅) corresponding to the values in **y**, and its entries may be denoted as *p*_*yk*_, with *k* ∈ {0, 1,…, *N*}.

In terms of look-up-tables entries, the learning relaxation index Δ*g*_*n*_ of definition (5) may be approximated as

(8) $$\Delta g_{n}\approx \overset{N}{\underset{k=0}{\sum }}\;|\;g_{n,k}-g_{n-1,k}\;|\;p_{y_{k}}\Delta_{y},\;n\geq 1.$$

### 2.3. Use of the neural system as generative model

When a suitable dual neural system described by the transference *g* (⋅) has been learnt, it may be effectively used to generate random samples drawn from the desired statistical distribution. The number of available input samples (that coincides with the number of output samples to be generated) is hereafter denoted by *Q*. The difficulty here is that the input samples *x* are known while the output samples *y* are supposed to be computed as *y* = *f*(*x*) . However, unlike in [10], the function *f* (⋅) is not known in the present setting as its inverse *g*(⋅) only has been learnt. Nevertheless, the inversion of function *g*(⋅) is not required in order to employ the dual neural system as a generative model, provided an appropriate usage of the look-up table representing *g*(⋅) is made. First, it is necessary to produce a realization {*x*_*i*_}, *i* ∈ {1,…, *Q*}, drawn from the available-generator distribution *p*_*x*_(⋅) (having, e.g., zero-mean Gaussian or uniform probability density function) ranging in *𝒳*. About generation of input samples, as they are generated by using an available generator whose range is wider than *𝒳*, some generated input samples will be necessarily discarded. The amount of discarded input samples may be quantified. Let us denote by *p*_*x*_(⋅) the cumulative distribution function of the input, namely,

(9) $$P_{x}(x)\overset{\text{def}}{=}\int_{-\infty }^{x}p_{x}(t)dt\;.$$

The ratio *ρ* of the number of discarded samples over the total number of generated samples is given by

(10) $$\rho (R_{x})\overset{\text{def}}{=}\frac{\text{discarded}\;\text{samples}}{\text{generated}\;\text{samples}}=1-2P_{x}(R_{x})\;.$$

The parameter *R*_*x*_ may thus be selected in order to adjust the value of *ρ*(*R*_*x*_) to design needs. Then, it is necessary to address the proper values in the learnt look-up table (**g, y**) ∈ ℝ^*N*+1^ × ℝ^*N*+1^ corresponding to the values of {*x*_*i*_}, *i* ∈ {1,…, *Q*}, by finding pointers *r*_*i*_ ∈ {0, 1,…,*N*} such that *g*_*r*_*i*__ ≈ *x*_*i*_. This means searching, in the whole look-up table, for the closest value of *g*(⋅) to the sample *x*_*i*_. Such operation should be performed in an efficient way. Finally, the desired set $\left\{{\overset{˜}{y}}_{i}\right\}$ of output samples, approximately distributed according to the probability density function *p*_*y*_(⋅), may be obtained by setting ${\overset{˜}{y}}_{i}:=y_{r_{i}},i\in \left\{1,\ldots ,Q\right\}$, where *y*_*r*_*i*__ denotes the *r*_*i*_th entry of the look-up table (**g, y**). (Commented MATLAB code is available on request.)

## 3. COMPUTER-BASED NUMERICAL EXPERIMENTS

In the following experiments, we consider generating random univariate samples with prescribed density function within prescribed ranges of interest, supposing that a prototype Gaussian random number generator is available. The prototype Gaussian distribution has zero mean and unitary variance. The parameter *R*_*x*_ was set to 1 in all the experiments, which corresponds to a ratio *ρ* ≈ 0.3173 that allows retaining about 70% of the generated input samples. The experiments were run on a 1.86 GHz, 512 MB-RAM platform.

### 3.1. Experiments on a “bi-Gaussian” distribution

The first case of generation of a random variable concerns a “bi-Gaussian” distribution defined by

(11) $$G_{2}(y)=\frac{1}{2}[\frac{1}{\sqrt{2\pi }\sigma_{1}}\exp(-\frac{(y-\mu_{1}{)}^{2}}{2\sigma_{1}^{2}})+\frac{1}{\sqrt{2\pi }\sigma_{2}}\exp\left(-\frac{(y-\mu_{2}{)}^{2}}{2\sigma_{2}^{2}}\right)]$$

that may assume fairly asymmetric shapes.

**Figure 3:**
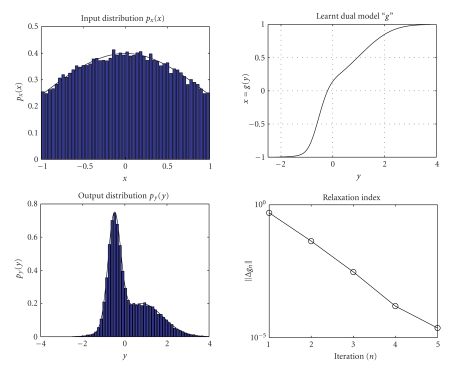
Result of dual neural system adaptation with Gaussian input and bi-Gaussian output.

The numerical results presented below pertain to values *σ*_1_ = 0.3, *μ*_1_ = −0.5, *σ*_2_ = 1 and *μ*_2_ = 0.8. The interval of interest for the output variable is set to *𝓎* = [−2.5 4]. The total number of generated output samples amounts to *Q* = 68219. The number of points in which the function *g*(⋅) is computed is *N* = 1000. The results obtained by running the learning algorithm (7) are shown in Figure 3. The values of the index Δ*g*_*n*_ shows that the fixed-point algorithm may be stopped after 5 iterations. In Figure 3, the histogram estimates (with 50 bins) of the generated Gaussian data and of the bi-Gaussian output—obtained with the learnt dual system—may be observed as well.

Cumulative results on repeated independent trials are illustrated. The number of iterations of the algorithm (7) was set to 10, while the other data stayed the same of the previous single-run experiment. The number *N* of points in which the function *g*(⋅) was computed ranged from 200 to 2000 with step 200, in order to obtain some information about the sensitivity of the algorithm to the selection of the number of points in the domain *𝓎* and about the influence of the number *N* in the computational complexity of the algorithm. In particular, the sensitivity of the algorithm with respect to the number *N* was measured via a discrepancy index DSC computed as follows. (a) The histogram-based estimate of the probability density function of the generated samples is computed on a number of bins equal to 50. The discrete values of such estimate are denoted by p^yb, *b* ∈ {1, 2,…, 50}. (b) The true values of the probability density function *p*_*y*_ (⋅) are computed in correspondence of the histogram's bin-centers. The discrete values of such probability density function are denoted by *p*_*yb*_, *b* ∈ {1, 2,…, 50}. (c) The weighted-square-difference-type discrepancy index is computed by the expression DSC=def∑b=150(p^yb−pyb)2pyb .

The average number of generated samples varies between about 68250 and 68290. The obtained results are summarized in Tables 1 and 2. The tables show the average run-time required for learning (expressed in seconds), the average run-time required to generate the samples (use of the learnt systems as a generative model) and average DSC index value. As it is readily appreciated, the computational complexity owing to the learning phase depends on the number of points used to approximate the nonlinear transference *g*(⋅) as expected, while the computational complexity owing to the generation phase depends only slightly on *N*. The sensitivity of the method measured by the discrepancy index DSC is high for low values of the parameter *N*, while it becomes quite low for values of *N* larger than 1000.

### 3.2. Experiments on a generalized Gaussian distribution

The second example of random samples generation is about a generalized Gaussian distribution [13]:

(12) $$\begin{array}{ll} T(y) & =\frac{{\text{sech}}^{\lambda -1}\left(y-\mu \right)-\lambda \,{\text{sech}}^{\lambda +1}\left(y-\mu \right)\sinh^{2}(y-\mu )}{\sqrt{2\pi }\sigma }\\ & \;\times \;\exp\left[-\frac{(\sinh\left(y\right){\sec\text{h}}^{\lambda }(y)-\sinh(\mu ){\sec\text{h}}^{\lambda }\left(\mu \right){)}^{2}}{2\sigma^{2}}\right], \end{array}$$

where sinh(⋅) denotes the hyperbolic sine function and sech(⋅) denotes the reciprocal of the hyperbolic cosine function (namely, the hyperbolic secant function) The present generalized Gaussian distribution (GGD) differs by the standard GGD model encountered in literature (see, e.g., [14]). It belongs to the general exponential family of distributions of the type *p*_*y*_(*y*) ∝ exp (−*κ* ^2^(*y*)), with *κ*(⋅) satisfying appropriate compatibility conditions. The distribution (12) as well as the GGD in [14] belong to the above exponential family.

**Figure 4:**
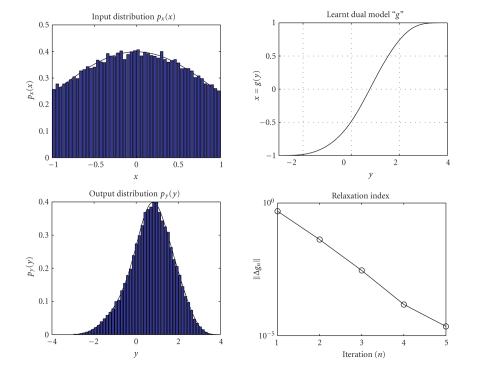
Result of dual neural system adaptation with Gaussian input and generalized Gaussian output.

The numerical results presented below pertain to values *σ* = 1, *μ* = 0.8, and *λ* = 0.5. The interval of interest for the output variable is set to *𝓎* = [−3 4]. The total number of generated output samples amounts to *Q* = 68335. The number of points in which the function *g*(⋅) is computed is *N* = 1000. The results obtained by running the learning algorithm (7) are shown in Figure 4. The values of the index Δ*g*_*n*_ show that the fixed-point algorithm may be safely stopped after 5 iterations again. In Figure 4, the histogram estimates (with 50 bins) of the generated Gaussian data and of the generalized Gaussian output may be observed as well.

Cumulative results are illustrated as well. The number of iterations of the algorithm (7) was set to 10, while the other data stayed the same of the previous single-run experiment. The number *N*of points ranged from 200 to 1000 with step 200. The average number of generated samples varies between about 68250 and 68290. The obtained results are summarized in Table 3.

### 3.3. Experiments on a Gamma distribution

The third example is repeated from [10]: we considered the generation of a (symmetric) Gamma distribution:

(13) $$B\left(y\right)\overset{\text{def}}{=}\frac{{\alpha \beta }^{1/\alpha }}{2\Gamma \left(1/\alpha \right)}\exp(-\beta \;|\;y{\;|}^{\alpha }).$$

This choice is motivated by the observation that the random number generation algorithm in [10] gives rise to the most inaccurate result when tested on the Gamma distribution .

**Figure 5:**
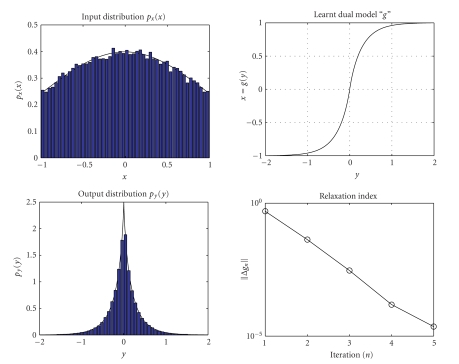
Result of dual neural system adaptation with Gaussian input and Gamma output.

The numerical results presented below pertain to values *α* = 0.8 and *β* = 4. The interval of interest for the output variable is set to *𝓎* = [−2 2]. The total number of generated output samples amounts to *Q* = 68355. The number of points in which the function *g*(⋅) is computed is *N* = 1500. The results obtained by running the learning algorithm (7) are shown in Figure 5. The values of the index Δ*g*_*n*_ show that the fixed-point algorithm may be safely stopped after 5 iterations again. Figure 5 shows the histogram estimates (with 50 bins) of the generated Gaussian data and of the Gamma-distributed output.

Cumulative results were obtained by setting the number of iterations of the algorithm (7) to 20, while the other data stayed the same of the previous single-run experiment. The number *N*of points ranged from 1000 to 1800 with step 200. The average number of generated samples varies between about 68230 and 68280. The obtained results are summarized in Table 4.

## 4. CONCLUSION

The aim of the present manuscript was to present a novel random number generation technique based on dual neural system learning. We elaborated over our recent work [10] in order to obtain a new learning algorithm free of the need of choosing parameters and normalization-criteria. The main idea is to shift the learning paradigm from the viewpoint of cardinal equation solving to dual cardinal equation solving, which appears to be more easily profitable.

The proposed numerical results confirmed the agreement between the desired and obtained distributions of the generated variate. The analysis of computational burden, in terms of running times, shows that the proposed algorithm is not computationally demanding.

## Figures and Tables

**Table 1:** Average results about the experiment on bi-Gaussian random number generation; averages computed over 100 independent trials when the algorithm (7) was iterated 10 times (first batch of results).

POINTS *N*	200	400	600	800	1000
AVG. LEARN. TIME	0.0092	0.0090	0.0109	0.0117	0.0133
AVG. GEN. TIME	0.0517	0.0514	0.0509	0.0508	0.0509
AVG. DSC	0.0026	0.0018	0.0011	0.0009	0.0008

**Table 2:** Average results about the experiment on bi-Gaussian random number generation; averages computed over 100 independent trials when the algorithm (7) was iterated 10 times (second batch of results).

POINTS *N*	1200	1400	1600	1800	2000
AVG. LEARN. TIME	0.0145	0.0155	0.0176	0.0197	0.0209
AVG. GEN. TIME	0.0527	0.0528	0.0527	0.0511	0.0523
AVG. DSC	0.0007	0.0006	0.0005	0.0006	0.0005

**Table 3:** Average results about the experiment on generalized Gaussian random number generation; averages computed over 100 independent trials when the algorithm (7) was iterated 10 times.

POINTS *N*	200	400	600	800	1000
AVG. LEARN. TIME	0.0129	0.0159	0.0229	0.0281	0.0320
AVG. GEN. TIME	0.0511	0.0517	0.0500	0.0502	0.0513
AVG. DSC	0.0038	0.0018	0.0011	0.0007	0.0005

**Table 4:** Average results about the experiment on Gamma random number generation; averages computed over 100 independent trials when the algorithm (7) was iterated 20 times.

POINTS N	1000	1200	1400	1600	1800
AVG. LEARN. TIME	0.0165	0.0176	0.0220	0.0242	0.0261
AVG. GEN. TIME	0.0516	0.0516	0.0504	0.0514	0.0513
AVG. DSC	0.0137	0.0118	0.0118	0.0109	0.0101

## References

[1] L'Ecuyer P, Banks J (1998). Random number generation. *The Handbook of Simulation*.

[2] Lagarias JC, Pomerance C (1990). Pseudorandom number generators in cryptography and number theory. *Cryptology and Computational Number Theory, Proceedings of Symposia in Applied Mathematics*.

[3] Niaki STA, Abbasi B NORTA and neural networks based method to generate RANDOM vectors with arbitrary marginal distributions and correlation matrix.

[4] Marsaglia G, Billard L (1985). A current view of random number generators. *Computer Science and Statistics: The Interface*.

[5] Niederreiter H (1992). *Random Number Generation and Quasi-Monte Carlo Methods*.

[6] Ripley BD (1990). Thoughts on pseudorandom number generators. *Journal of Computational and Applied Mathematics*.

[7] Cichocki A, Amari S-I (2002). *Adaptive Blind Signal and Image Processing: Learning Algorithms and Applications*.

[8] Knuth DE (1997). *The Art of Computer Programming: Seminumerical Algorithms*.

[9] Papoulis A (1996). *Probability and Statistics*.

[10] Fiori S (2006). Neural systems with numerically-matched input-output statistic: variate generation. *Neural Processing Letters*.

[11] Fiori S (2007). Neural systems with numerically matched input-output statistic: isotonic bivariate statistical modeling. *Computational Intelligence and Neuroscience*.

[12] Ghosh S, Henderson SG, Yücesan E, Chen C-H, Snowdon JL, Charnes JM Properties of the NORTA method in higher dimensions.

[13] Tsai AC (2006).

[14] Kokkinakis K, Nandi AK (2005). Exponent parameter estimation for generalized Gaussian probability density functions with application to speech modeling. *Signal Processing*.

